# Supplementary material for: Assessing the Intestinal Permeability and Anti-Inflammatory Potential of Sesquiterpene Lactones from Chicory
Source: Nutrients. 2020 Nov 19;12(11):3547. doi: 10.3390/nu12113547 (PMC7699524; doi:10.3390/nu12113547)
Supplement: Supplementary file 1 [file nutrients-12-03547-s001.pdf]

# Supplementary file

**Table S1.** Optimization of LC-MS detection and quantification of SLs based upon the calibration curves obtained in a sample-matched matrix (HBSS buffer), with definition of range of linearity, limit of detection (LOD) and limit of quantification (LOQ).

| Compound | RT (min) [a] | *m/z* [b] | $R^2$ [c] | Range (nM) | LOD (nM) [d] | LOQ (nM) [e] | RSD (QC) (%) [f] |
|---|---|---|---|---|---|---|---|
| Costunolide [M+H] | 31.30 | 233.153 | 0.999 | 25,000–12.2 | 20.9 | 69.9 | 1.85 |
| Parthenolide [M+H]-H20 | 27.44 | 231.137 | 0.999 | 25,000–3.0 | 15.5 | 51.6 | 4.52 |
| Lactucin [M+H] | 13.82 | 277.106 | 0.995 | 25,000–3.0 | 10.1 | 33.8 | 3.27 |
| Lactucopicrin [M+H] | 22.20 | 411.142 | 0.993 | 25,000–6.1 | 13.6 | 45.5 | 3.95 |
| 11β,13-dihydrolactucin [M+H] | 13.07 | 279.122 | 0.996 | 25,000–97.7 | 119.5 | 398.2 | 3.60 |
| 11β,13-dihydrolactucopicrin [M+H] | 22.16 | 413.158 | 0.993 | 25,000–24.41 | 22.4 | 74.5 | 5.37 |

[a] RT: Retention time in minutes. [b] *m/z*: Mass: charge ratio. [c] $R^2$: Coefficient of determination. [d] LOD: Limit of detection. [e] LOQ: Limit of quantification. [f] RSD: Relative standard deviation (QC Quality control sample—a pool of all experimental samples). A pooled sample of all experimental samples (a QC) was created to check for method precision. Although each SL is diluted within the pooled QC, the levels of precision still fell within 1.8-5.4% RSD.

**A**

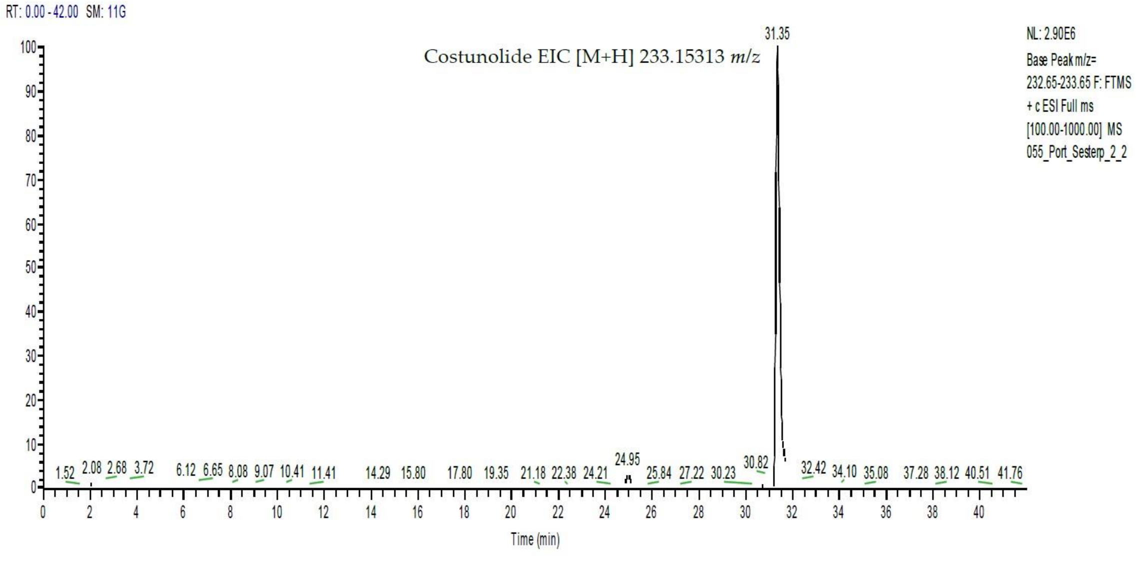

**B**

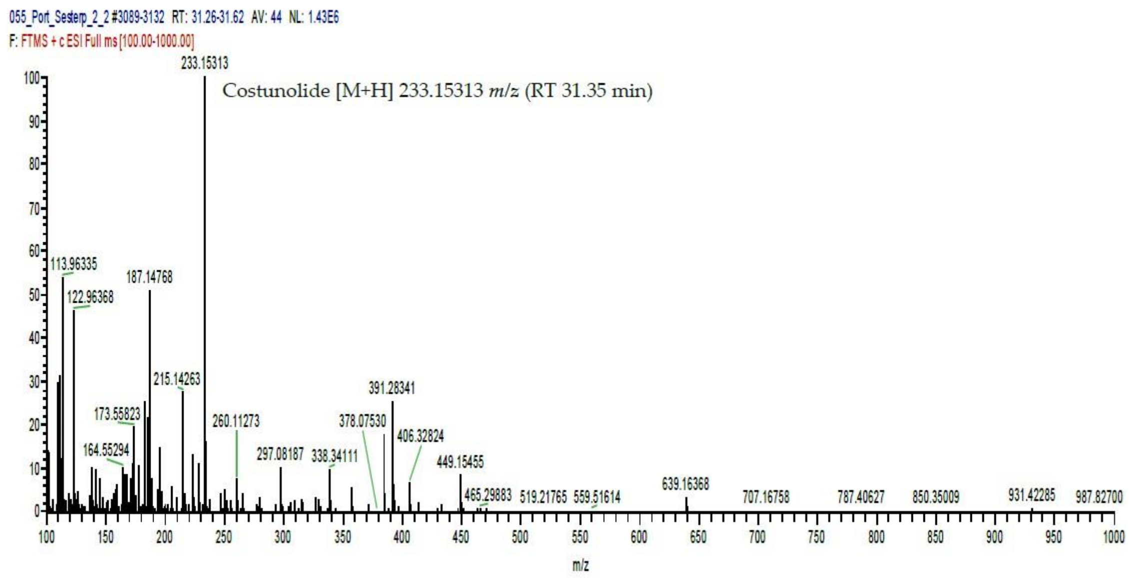


Costunolide-cysteine conjugate, in-source fragment [M+Cys+H]-Cys 233.15313 *m/z*

**C**

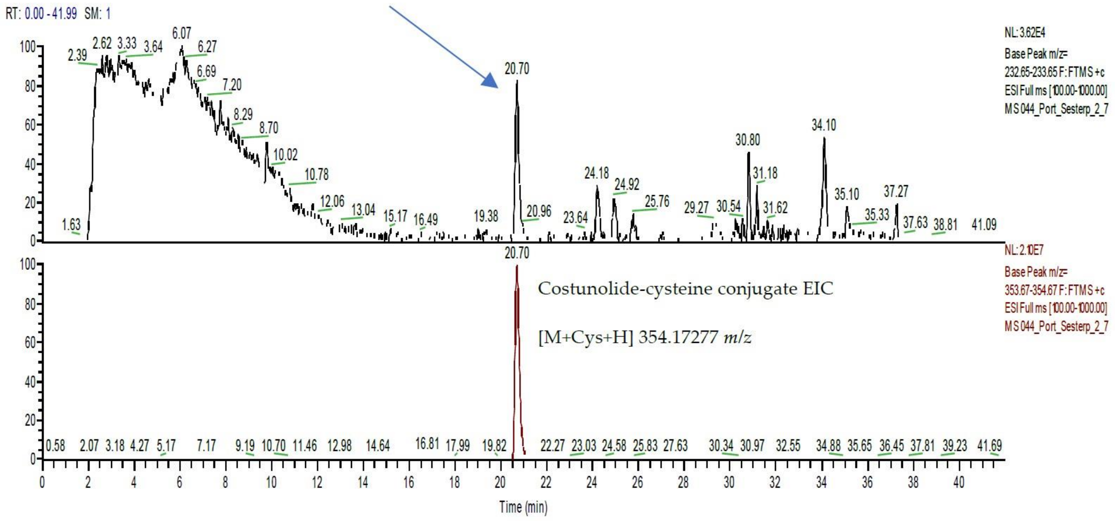


**D**

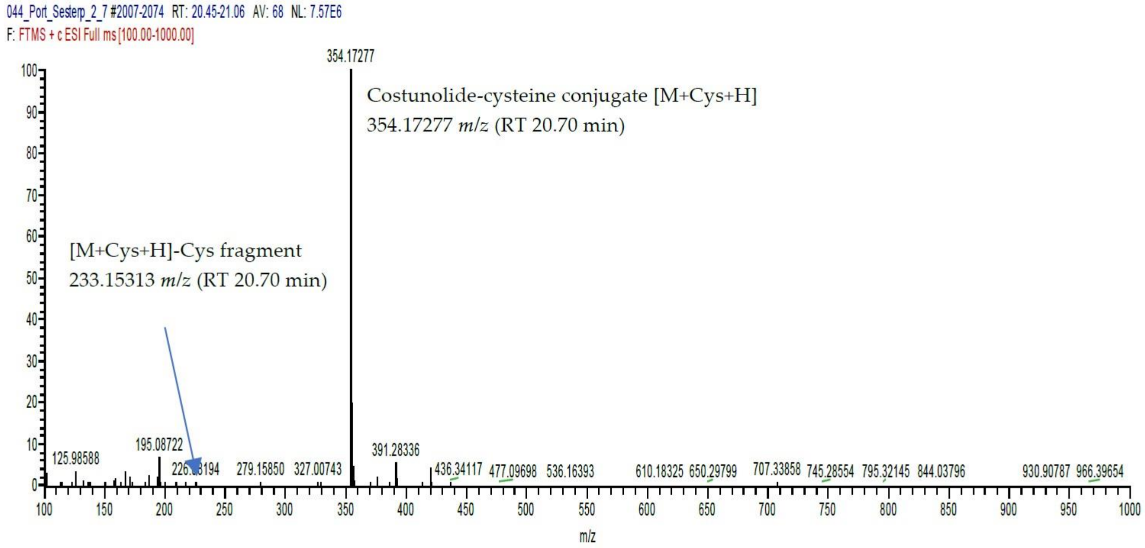

**Figure S1.** LC-MS analysis of costunolide uptake from 0 hours to 4 hours in the apical side. A) 10 μM costunolide in HBSS media (time 0 hours): Extracted Ion Chromatogram (EIC) costunolide ([M+H] *m/z* 233.15313 RT 31.35 minutes); B) 10 μM costunolide in HBSS media (time 0 hours): costunolide Electrospray Ionisation (ESI) positive mode mass spectrum ([M+H] *m/z* 233.15313 RT 31.35 minutes); C) Costunolide apical side (time 4 hours): Extracted Ion Chromatogram (EIC) costunolide [M+H] (*m/z* 233.15313) (upper panel); EIC costunolide-cysteine bound [M+Cys+H] (*m/z* 354.17277) (lower panel). Note in upper panel that the costunolide peak previously detected at RT 31.35 minutes in (A) is no longer detected. Binding to cysteine results in a RT shift to 20.70 minutes; due to ESI in-source fragmentation cysteine is dissociated from costunolide, resulting in the detection of *m/z* 233.15313 [M+Cys+H]-Cys at RT 20.70 minutes (upper panel); D) Costunolide apical side (time 4 hours): costunolide-cysteine bound ESI positive mode mass spectrum ([M+Cys+H] *m/z* 354.17277 RT 20.70 minutes). Note minor peak detected for ESI in-source fragment ([M+Cys+H]-Cys fragment 233.15313 *m/z* RT 20.70 minutes).

**A**

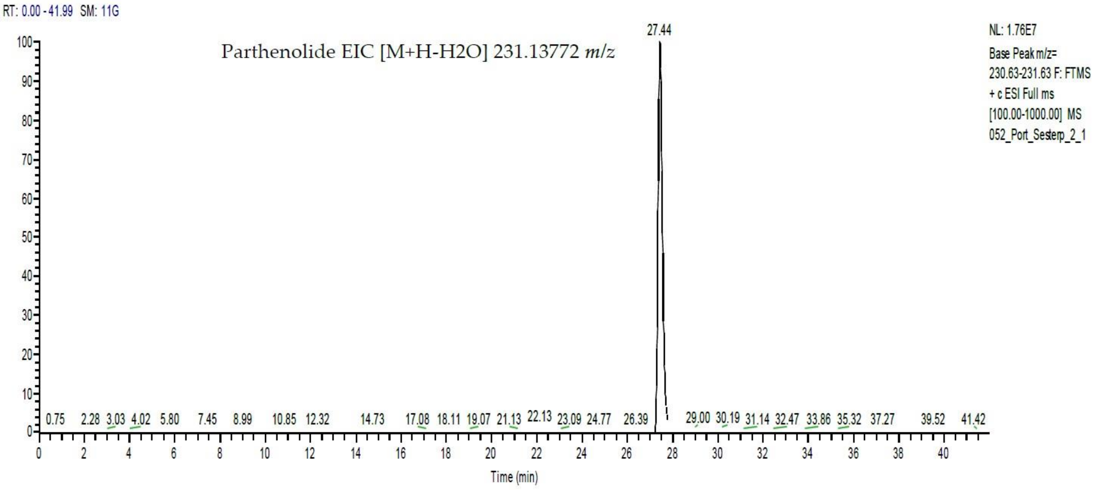


**B**

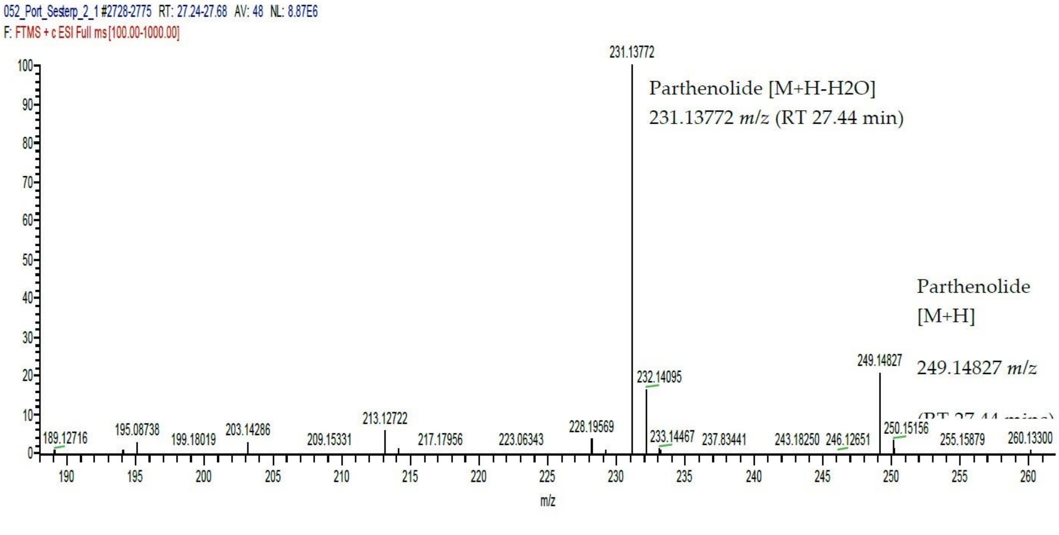


Parthenolide-cysteine conjugate, in-source fragment [M+Cys+H]-Cys-H2O 231.13772 *m/z*

C

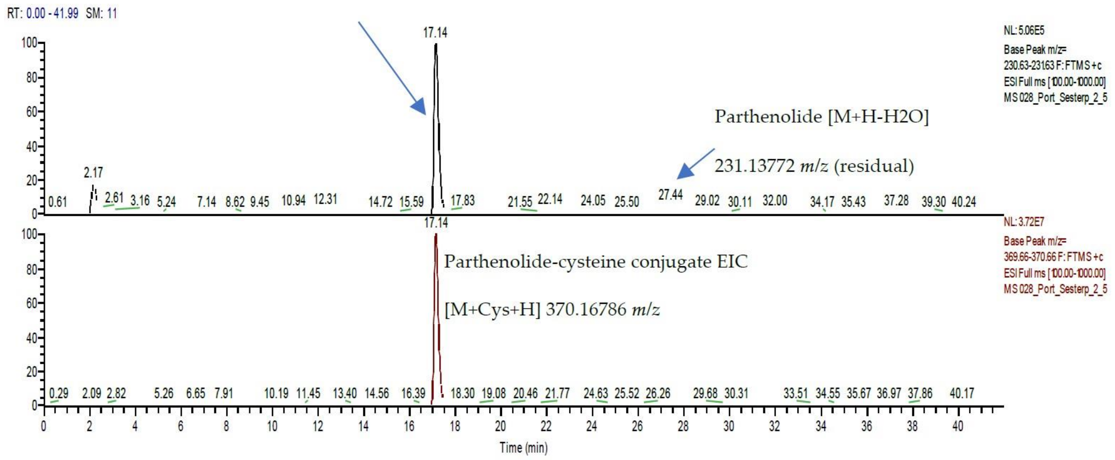


D

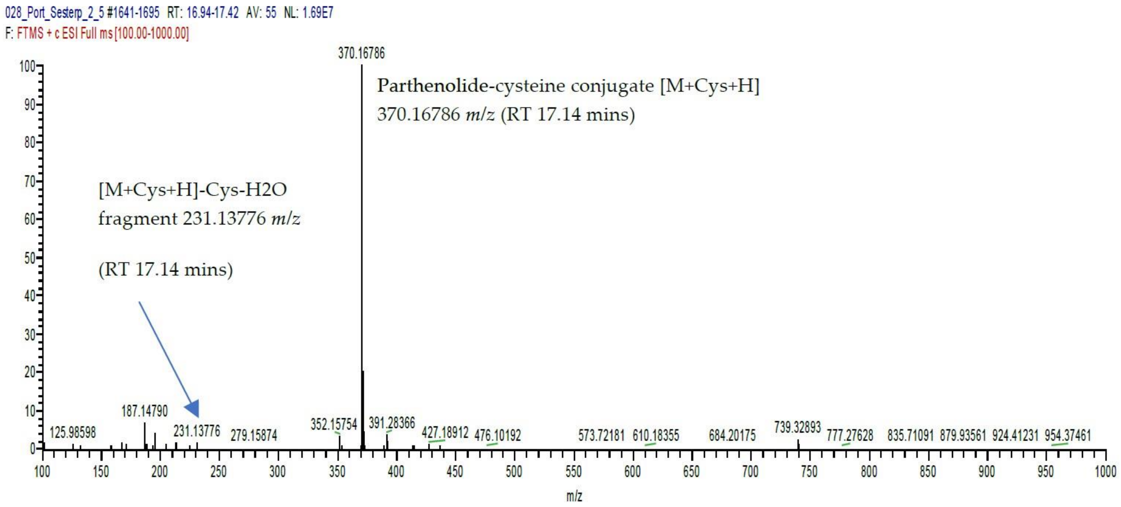


**Figure S2.** LC-MS analysis of parthenolide uptake from 0 hours to 4 hours in the apical side. A) 10 μM parthenolide in HBSS media (time 0 hours): EIC Parthenolide ([M+H]-H2O *m/z* 231.13772 RT 27.44 minutes); B) 10 μM parthenolide in HBSS media (time 0 hours): parthenolide ESI positive mode mass spectrum ([M+H]-H2O *m/z* 231.13772 RT 27.44 minutes; [M+H] *m/z* 249.14872 RT 27.44 minutes); C) Parthenolide apical side (time 4 hours): Extracted Ion Chromatogram (EIC) parthenolide [M+H]-H2O (*m/z* 231.13772) (upper panel); EIC parthenolide-cysteine bound [M+Cys+H] (*m/z* 370.16786) (lower panel). Note in upper panel that the parthenolide peak previously detected at RT 27.44 minutes in (A) is only detected at low residual levels. Binding to cysteine results in a RT shift to 17.14 minutes; due to ESI in-source fragmentation cysteine is dissociated from parthenolide, resulting in the detection of *m/z* 231.13772 [M+Cys+H]-Cys-H2O at RT 17.14 minutes (upper panel); D) Parthenolide apical side (time 4 hours): parthenolide-cysteine bound ESI positive mode mass spectrum ([M+Cys+H] *m/z* 370.16786 RT 17.14 minutes). Note minor peak detected for ESI in-source fragment ([M+Cys+H]-Cys-H2O fragment 231.13776 *m/z* RT 17.14 minutes).

**A**

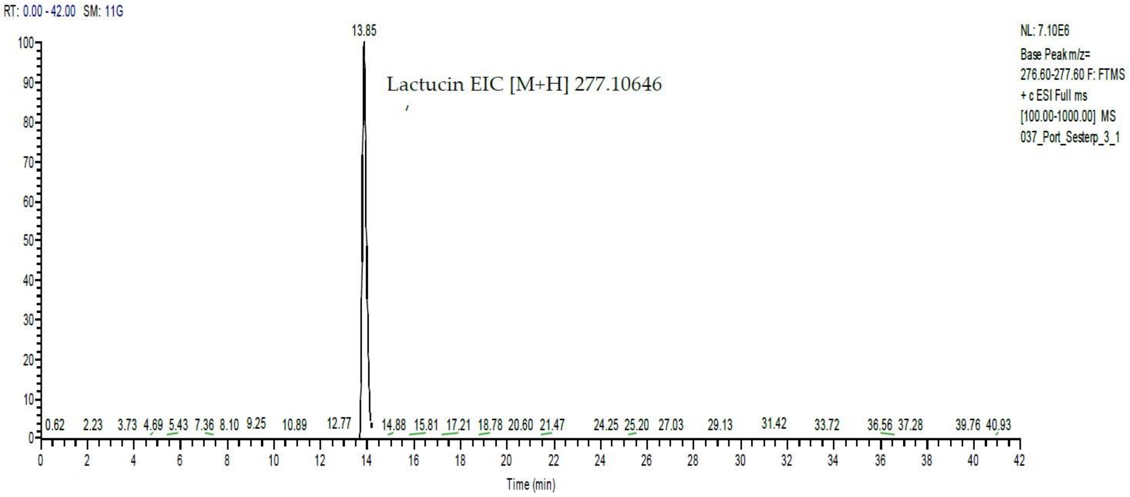
RT: 0.00 - 42.00 SM: 11G
13.85
Lactucin EIC [M+H] 277.10646
NL: 7.10E6
Base Peak m/z=
276.60-277.60 F: FTMS
+ c ESI Full ms
[100.00-1000.00] MS
037_Port_Sesterp_3_1
0.62 2.23 3.73 4.69 5.43 7.36 8.10 9.25 10.89 12.77 14.88 15.81 17.21 18.78 20.60 21.47 24.25 25.20 27.03 29.13 31.42 33.72 36.56 37.28 39.76 40.93
Time (min)

**B**

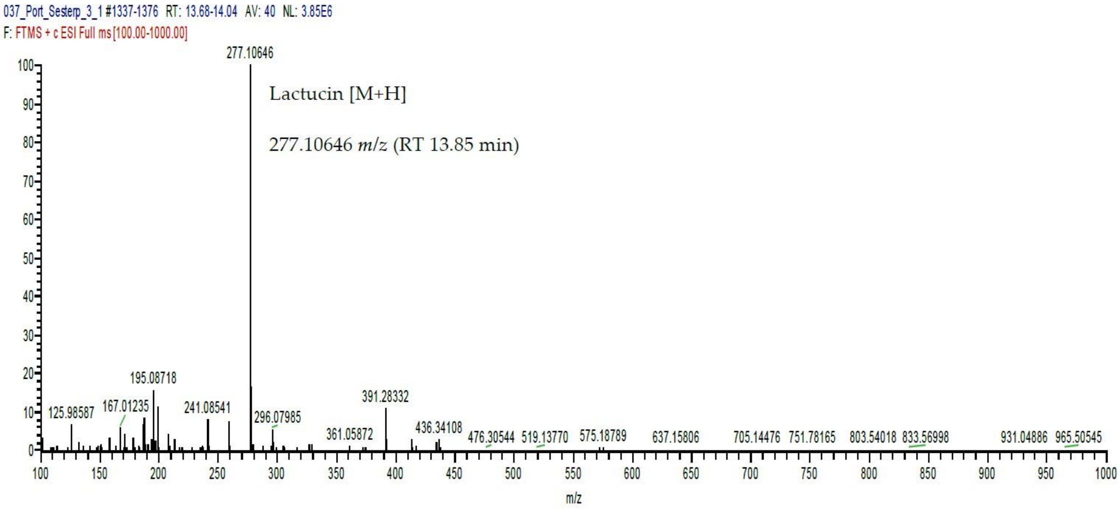
037_Port_Sesterp_3_1 #1337-1376 RT: 13.68-14.04 AV: 40 NL: 3.85E6
F: FTMS + c ESI Full ms [100.00-1000.00]
277.10646
Lactucin [M+H]
277.10646 m/z (RT 13.85 min)
125.98587 167.01235 195.08718 241.08541 296.07985 361.05872 391.28332 436.34108 476.30544 519.13770 575.18789 637.15806 705.14476 751.78165 803.54018 833.56998 931.04886 965.50545
m/z

C

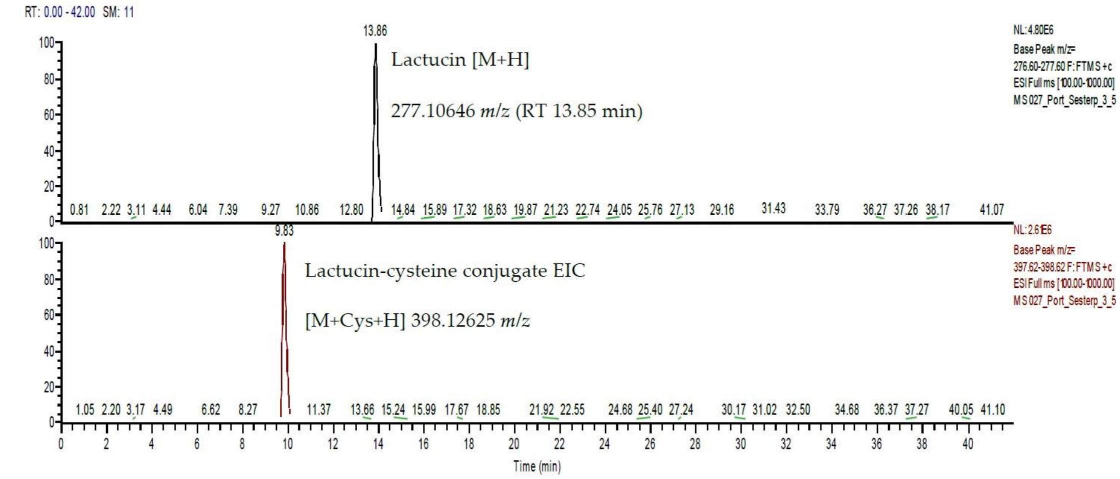


D

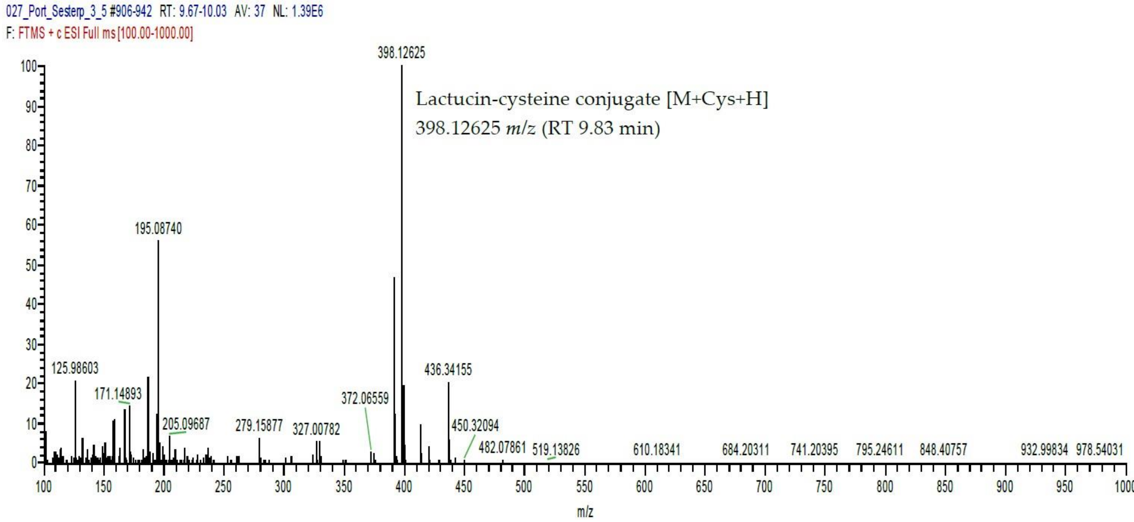


**Figure S3.** LC-MS analysis of lactucin uptake from 0 hours to 4 hours in the apical side. A) 10 µM lactucin in HBSS media (time 0 hours): EIC lactucin ([M+H] *m/z* 277.10646 RT 13.85 minutes); B) 10 µM lactucin in HBSS media (time 0 hours): lactucin ESI positive mode mass spectrum ([M+H] *m/z* 277.10646 RT 13.85 minutes); C) Lactucin apical side (time 4 hours): Extracted Ion Chromatogram (EIC) lactucin [M+H] (*m/z* 277.10646) (upper panel); EIC lactucin-cysteine bound [M+Cys+H] (*m/z* 398.12625) (lower panel). Note in upper panel that the lactucin peak previously detected at RT 13.85 minutes in (A) is still detected at high intensity at RT 13.85 minutes due to the level of lactucin uptake being low. Binding to cysteine results in a RT shift to 9.83 minutes; since lactucin is only up-taken at a low level, the ESI in-source fragmentation of cysteine (*m/z* 277.10646 RT 9.83 minutes) is not observed due to being below the MS limit of detection; D) Lactucin apical side (time 4 hours): lactucin-cysteine bound ESI positive mode mass spectrum ([M+Cys+H] *m/z* 398.12625 RT 9.83 minutes). Note that since lactucin is only up-taken at a low level, the ESI in-source fragmentation of cysteine (([M+Cys+H]-Cys fragment *m/z* 277.10646 RT 9.83 minutes) is not observed due to being below the mass spectrometers limit of detection.

A

B

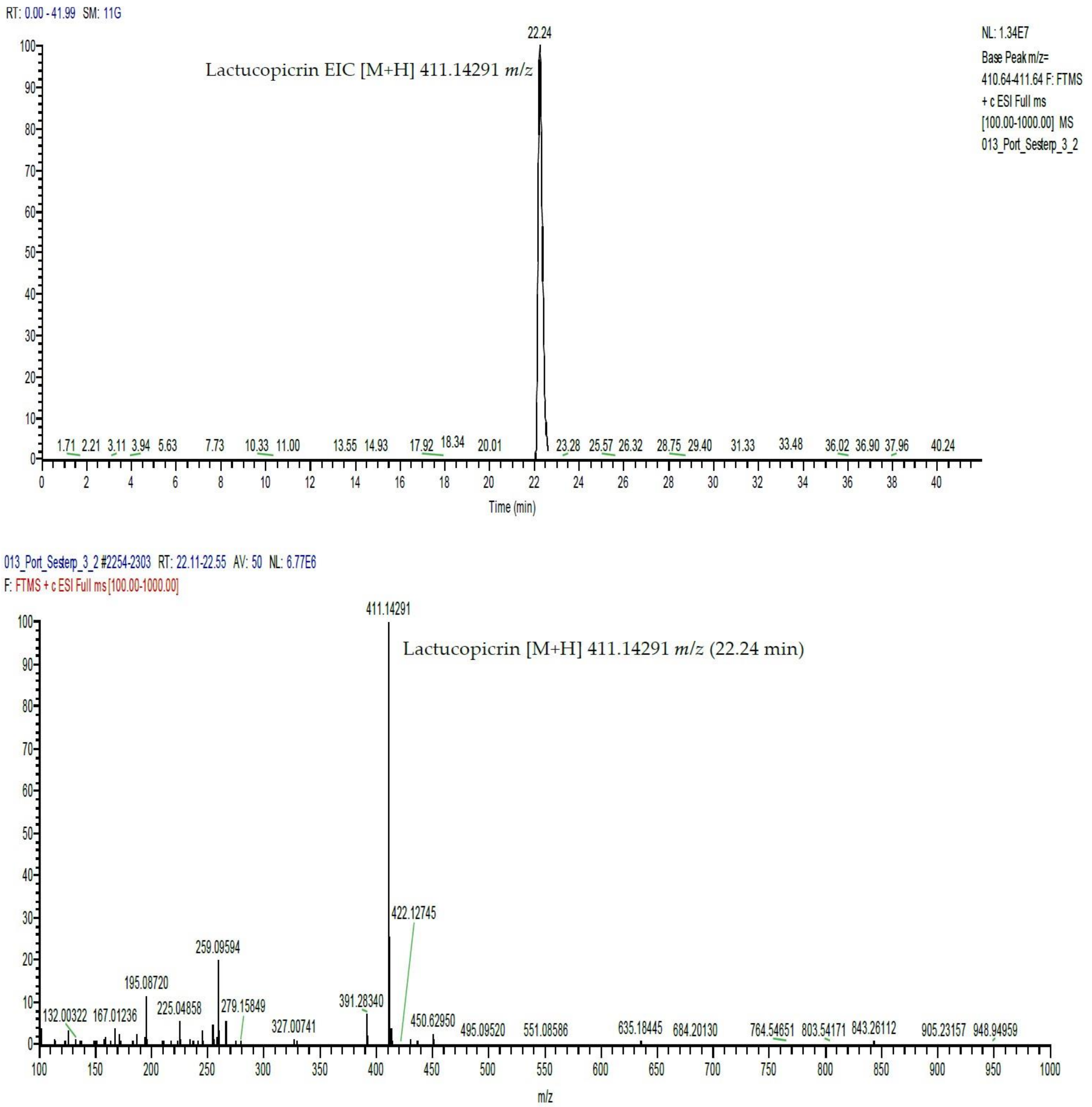

RT: 0.00 - 41.99 SM: 11G
22.24
Lactucopicrin EIC [M+H] 411.14291 *m/z*
NL: 1.34E7
Base Peak m/z=
410.64-411.64 F: FTMS
+ c ESI Full ms
[100.00-1000.00] MS
013_Port_Sesterp_3_2
1.71 2.21 3.11 3.94 5.63 7.73 10.33 11.00 13.55 14.93 17.92 18.34 20.01 23.28 25.57 26.32 28.75 29.40 31.33 33.48 36.02 36.90 37.96 40.24
Time (min)
013_Port_Sesterp_3_2 #2254-2303 RT: 22.11-22.55 AV: 50 NL: 6.77E6
F: FTMS + c ESI Full ms [100.00-1000.00]
411.14291
Lactucopicrin [M+H] 411.14291 *m/z* (22.24 min)
422.12745
259.09594
195.08720
391.28340
132.00322 167.01236 225.04858 279.15849 327.00741 450.62950 495.09520 551.08586 635.18445 684.20130 764.54651 803.54171 843.26112 905.23157 948.94959
m/z

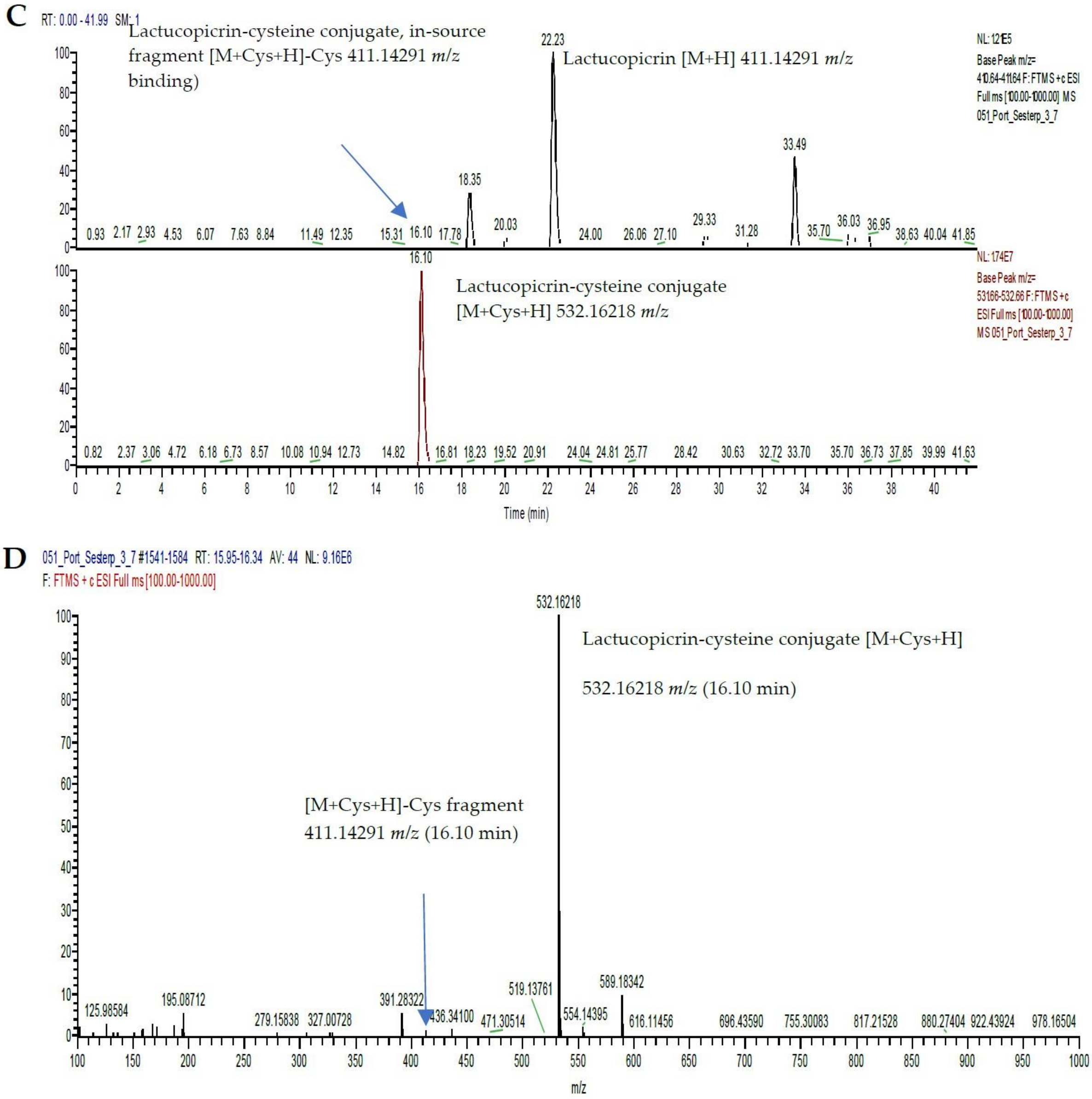


**Figure S4.** LC-MS analysis of lactucopicrin uptake from 0 hours to 4 hours in the apical side. A) 10 µM lactucopicrin in HBSS media (time 0 hours): Extracted Ion Chromatogram (EIC) lactucopicrin ([M+H] *m/z* 411.14291 RT 22.24 minutes); B) 10 µM lactucopicrin in HBSS media (time 0 hours): lactucopicrin Electrospray Ionisation (ESI) positive mode mass spectrum ([M+H] *m/z* 411.14291 RT 22.24 minutes); C) Lactucopicrin apical side (time 4 hours): Extracted Ion Chromatogram (EIC) lactucopicrin ([M+H] *m/z* 411.14291 (upper panel); EIC Lactucopicrin-Cysteine bound [M+Cys+H] (*m/z* 532.16218) (lower panel). Note in upper panel that the lactucopicrin peak previously detected at RT 22.24 minutes in (A) is still detected at slightly lower levels. Binding to cysteine results in a RT shift to 16.10 minutes (lower panel), due to ESI in-source fragmentation cysteine is dissociated from lactucopicrin, resulting in the detection of a residual chromatographic peak for *m/z* 411.14291 [M+Cys+H]-Cys at RT 16.10 minutes (upper panel); D) lactucopicrin apical side (time 4 hours): lactucopicrin-cysteine bound ESI positive mode mass spectrum ([M+Cys+H] *m/z* 532.16218 RT 16.10 minutes). Note minor peak detected for ESI in-source fragment ([M+Cys+H]-Cys fragment 411.14291 *m/z* RT 16.10 minutes).
